# Author Correction: A map of the rubisco biochemical landscape

**DOI:** 10.1038/s41586-025-08707-7

**Published:** 2025-02-10

**Authors:** Noam Prywes, Naiya R. Phillips, Luke M. Oltrogge, Sebastian Lindner, Leah J. Taylor-Kearney, Yi-Chin Candace Tsai, Benoit de Pins, Aidan E. Cowan, Hana A. Chang, Renée Z. Wang, Laina N. Hall, Daniel Bellieny-Rabelo, Hunter M. Nisonoff, Rachel F. Weissman, Avi I. Flamholz, David Ding, Abhishek Y. Bhatt, Oliver Mueller-Cajar, Patrick M. Shih, Ron Milo, David F. Savage

**Affiliations:** 1 Innovative Genomics Institute, University of California Berkeley, Berkeley, CA USA; 2 Howard Hughes Medical Institute, University of California Berkeley, Berkeley, CA USA; 3 Department of Molecular and Cell Biology, University of California Berkeley, Berkeley, CA USA; 4 University of Heidelberg, Heidelberg, Germany; 5 Department of Plant and Microbial Biology, University of California Berkeley, Berkeley, CA USA; 6 School of Biological Sciences, Nanyang Technological University, Singapore, Singapore; 7 Department of Biology, University of Naples Federico II, Naples, Italy; 8 Joint BioEnergy Institute, Lawrence Berkeley National Laboratory, Emeryville, CA USA; 9 Biophysics, University of California Berkeley, Berkeley, CA USA; 10 California Institute for Quantitative Biosciences (QB3), University of California Berkeley, Berkeley, CA USA; 11 Center for Computational Biology, University of California Berkeley, Berkeley, CA USA; 12 Division of Biology and Biological Engineering, California Institute of Technology, Pasadena, CA USA; 13 School of Medicine, University of California San Diego, La Jolla, CA USA; 14 Environmental Genomics and Systems Biology Division, Lawrence Berkeley National Laboratory, Berkeley, CA USA; 15 Feedstocks Division, Joint BioEnergy Institute, Emeryville, CA USA; 16 Department of Plant and Environmental Sciences, Weizmann Institute of Science, Rehovot, Israel

**Keywords:** Rubisco, Enzymes

Correction to: *Nature* 10.1038/s41586-024-08455-0 Published online 22 January 2025

In the version of the article initially published, the affiliations of Hana A. Chang (Department of Plant and Microbial Biology, University of California Berkeley, Berkeley, CA, USA) and Ron Milo (Department of Plant and Environmental Sciences, Weizmann Institute of Science, Rehovot, Israel) were incorrect and have now been amended in the HTML and PDF versions of the article.

