# Supplementary material for: A map of the rubisco biochemical landscape
Source: Nature. 2025 Jan 22;638(8051):823–8. doi: 10.1038/s41586-024-08455-0 (PMC11839469; doi:10.1038/s41586-024-08455-0)
Supplement: Supplementary file 1 — Reporting Summary [file 41586_2024_8455_MOESM1_ESM.pdf]

nature portfolio

Corresponding author(s): David Savage

Last updated by author(s): Oct 31, 2024

Reporting Summary

Nature Portfolio wishes to improve the reproducibility of the work that we publish. This form provides structure for consistency and transparency in reporting. For further information on Nature Portfolio policies, see our Editorial Policies and the Editorial Policy Checklist.

Statistics

For all statistical analyses, confirm that the following items are present in the figure legend, table legend, main text, or Methods section.

n/a	Confirmed	
☐	☒	The exact sample size (*n*) for each experimental group/condition, given as a discrete number and unit of measurement
☐	☒	A statement on whether measurements were taken from distinct samples or whether the same sample was measured repeatedly
☐	☒	The statistical test(s) used AND whether they are one- or two-sided<br>*Only common tests should be described solely by name; describe more complex techniques in the Methods section.*
☐	☒	A description of all covariates tested
☐	☒	A description of any assumptions or corrections, such as tests of normality and adjustment for multiple comparisons
☐	☒	A full description of the statistical parameters including central tendency (e.g. means) or other basic estimates (e.g. regression coefficient) AND variation (e.g. standard deviation) or associated estimates of uncertainty (e.g. confidence intervals)
☐	☒	For null hypothesis testing, the test statistic (e.g. *F*, *t*, *r*) with confidence intervals, effect sizes, degrees of freedom and *P* value noted<br>*Give P values as exact values whenever suitable.*
☒	☐	For Bayesian analysis, information on the choice of priors and Markov chain Monte Carlo settings
☒	☐	For hierarchical and complex designs, identification of the appropriate level for tests and full reporting of outcomes
☐	☒	Estimates of effect sizes (e.g. Cohen's *d*, Pearson's *r*), indicating how they were calculated

Our web collection on statistics for biologists contains articles on many of the points above.

Software and code

Policy information about availability of computer code

Data collection	HMMER 3.4
Data analysis	All data analysis components can be found at https://github.com/SavageLab/reads_processing<br>Packages used:<br>pandas 2.2.1<br>matplotlib 3.8.4<br>Biopython 1.81<br>numpy 1.26.4<br>scipy 1.12.0<br>seaborn 0.12.2<br>sklearn 1.2.2<br>pysam 0.21.0<br>Samtools 1.21

For manuscripts utilizing custom algorithms or software that are central to the research but not yet described in published literature, software must be made available to editors and reviewers. We strongly encourage code deposition in a community repository (e.g. GitHub). See the Nature Portfolio guidelines for submitting code & software for further information.

Data

Policy information about availability of data

All manuscripts must include a data availability statement. This statement should provide the following information, where applicable:

- Accession codes, unique identifiers, or web links for publicly available datasets
- A description of any restrictions on data availability
- For clinical datasets or third party data, please ensure that the statement adheres to our policy

Sequences for our form II rubisco phylogeny were assembled from UniRef100
Our raw sequencing reads can be accessed on the NCBI SRA, accession ID: PRJNA1181558
All other data are available in the main text or the supplementary materials.

Research involving human participants, their data, or biological material

Policy information about studies with human participants or human data. See also policy information about sex, gender (identity/presentation), and sexual orientation and race, ethnicity and racism.

Reporting on sex and gender	N/A
Reporting on race, ethnicity, or other socially relevant groupings	N/A
Population characteristics	N/A
Recruitment	N/A
Ethics oversight	N/A

Note that full information on the approval of the study protocol must also be provided in the manuscript.

Field-specific reporting

Please select the one below that is the best fit for your research. If you are not sure, read the appropriate sections before making your selection.

☒ Life sciences ☐ Behavioural & social sciences ☐ Ecological, evolutionary & environmental sciences

For a reference copy of the document with all sections, see nature.com/documents/nr-reporting-summary-flat.pdf

Life sciences study design

All studies must disclose on these points even when the disclosure is negative.

Sample size	Triplicate or greater as indicated in figure panels. The only exception is the in vitro work in Extended Data figure 6B (grey points indicated in the figure are not repeated). Sample sizes were not chosen based on a calculation. Our default value for replication was triplicate. In the case of the experiment that generated the most critical dataset (figure 1G) we performed 9 replicates.
Data exclusions	No data was excluded, all data is available in the supplementary files.
Replication	All attempts at replication were successful.
Randomization	No randomization was used since it was not appropriate for this study, all analyses were done programmatically.
Blinding	Blinding was not relevant to our study, all analyses were done programmatically.

Reporting for specific materials, systems and methods

We require information from authors about some types of materials, experimental systems and methods used in many studies. Here, indicate whether each material, system or method listed is relevant to your study. If you are not sure if a list item applies to your research, read the appropriate section before selecting a response.

Materials & experimental systems

n/a	Involved in the study
☐	☒ Antibodies
☒	☐ Eukaryotic cell lines
☒	☐ Palaeontology and archaeology
☒	☐ Animals and other organisms
☒	☐ Clinical data
☒	☐ Dual use research of concern
☒	☐ Plants

Methods

n/a	Involved in the study
☒	☐ ChIP-seq
☒	☐ Flow cytometry
☒	☐ MRI-based neuroimaging

Antibodies

Antibodies used

Polyclonal Rabbit Anti-RbcL II, Agrisera, AS15 2955, Lot 2111
Polyclonal Goat to Rabbit IgG, abcam, ab205718, Lot GR3366929-1
Monoclonal clone 8E2/2, Mouse anti-DnaK, abcam, ab69617, 103741-3
Donkey anti-Mouse IgG-HRP, Santa Cruz BioTechnology, sc-2314, Lot C2012

Validation

The anti-rbcL II antibody was validated against Alexandrium catenella, Amphidinium carterae, Chaetoceros neogracilis, Rhodobacter capsulatus, Rhodospirillum rubrum (relevant to this study)
Cho et al. (2021). SxtA localizes to chloroplasts and changes to its 3'UTR may reduce toxin biosynthesis in non-toxic Alexandrium catenella (Group I). Harmful Algae, 2021,101972,ISSN 1568-9883, https://doi.org/10.1016/j.hal.2020.101972. Immunolocalization
Bausch et al. (2019). Combined effects of simulated acidification and hypoxia on the harmful dinoflagellate Amphidinium carterae. Marine Biology, June 2019, 166:80.
Long et al. (2018). Carboxysome encapsulation of the CO2-fixing enzyme Rubisco in tobacco chloroplasts. Nat Commun. 2018 Sep 3;9(1):3570. doi: 10.1038/s41467-018-06044-0.

The anti-DnaK antibody has been used in 37 citations. The manufacturer states:
Mouse Monoclonal DNAK antibody. Suitable for WB and reacts with Recombinant full length protein - Escherichia coli, Escherichia coli samples. Cited in 37 publications. Immunogen corresponding to Full Length Protein corresponding to Escherichia coli K-12 dnaK.
The antibody has been validated against E. coli DnaK which is relevant to this study.

Plants

Seed stocks

N/A

Novel plant genotypes

N/A

Authentication

N/A
