# Supplementary figures and images for: A map of the rubisco biochemical landscape
Source: Nature. 2025 Jan 22;638(8051):823–8. doi: 10.1038/s41586-024-08455-0 (PMC11839469; doi:10.1038/s41586-024-08455-0)

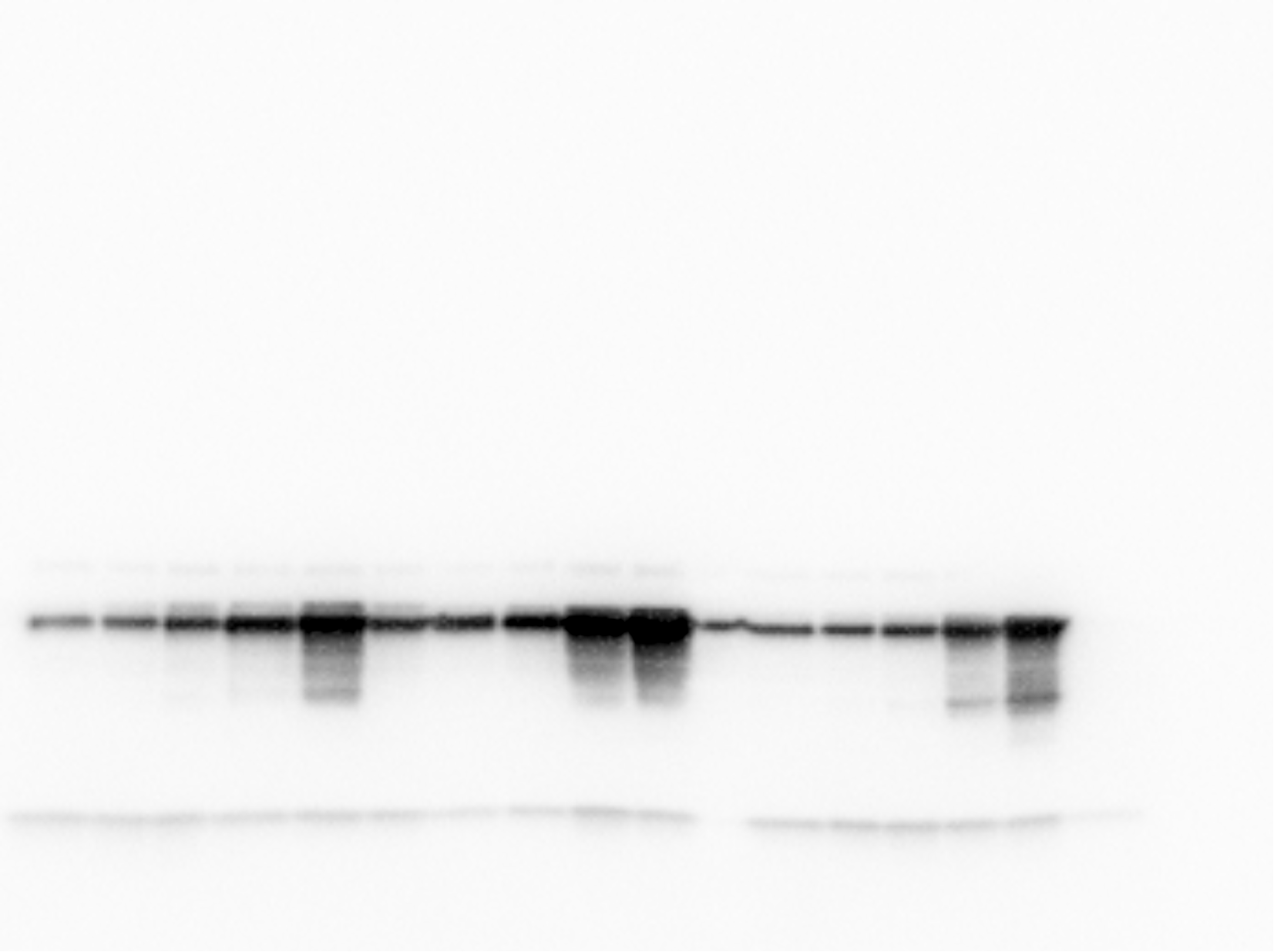

Supplement: Supplementary file 6 — Unedited western blot images. [file 41586_2024_8455_MOESM6_ESM.zip › Raw western images/2024-01-18-6.tif]

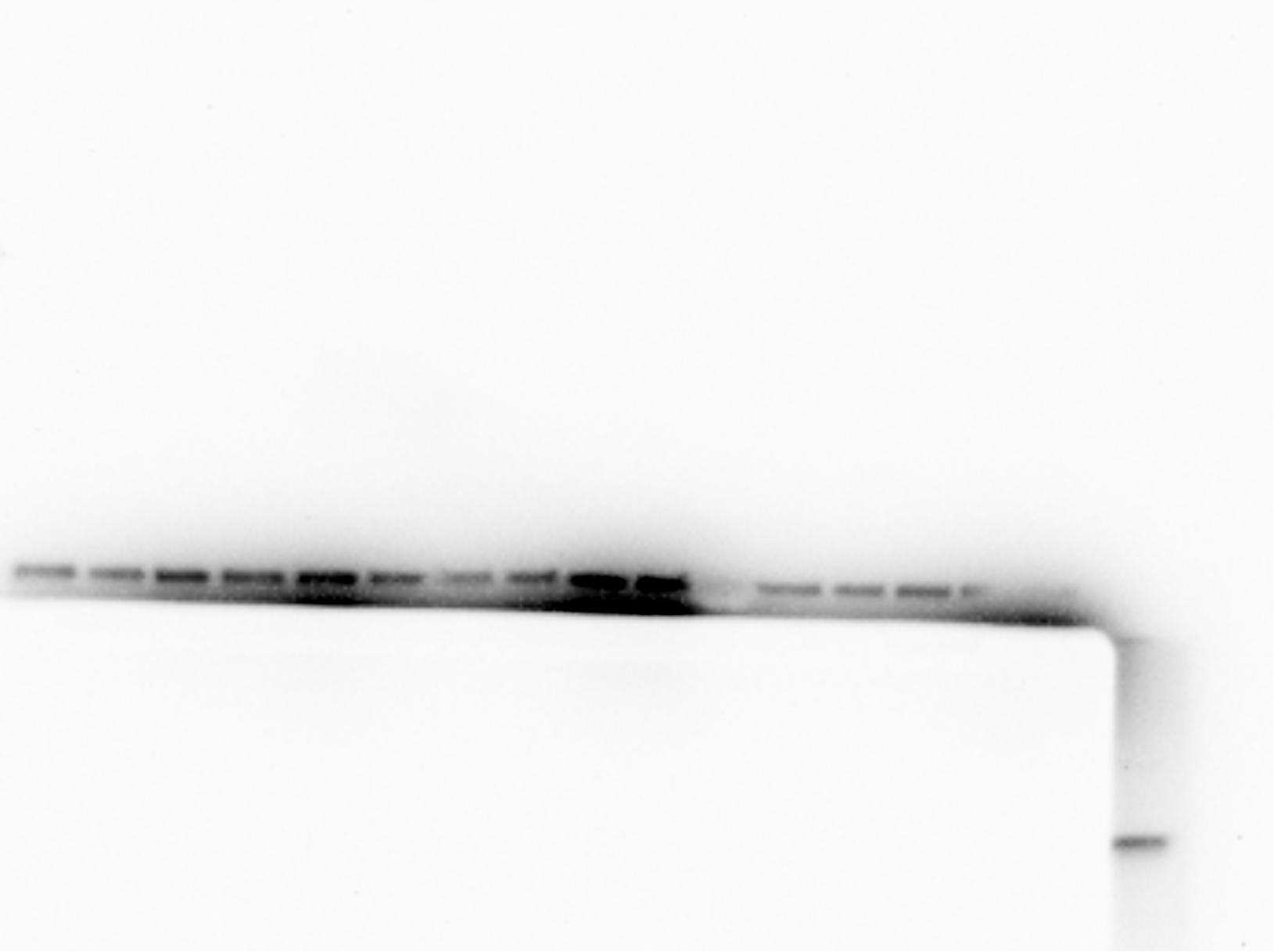

Supplement: Supplementary file 6 — Unedited western blot images. [file 41586_2024_8455_MOESM6_ESM.zip › Raw western images/2024-01-18-4.tif]

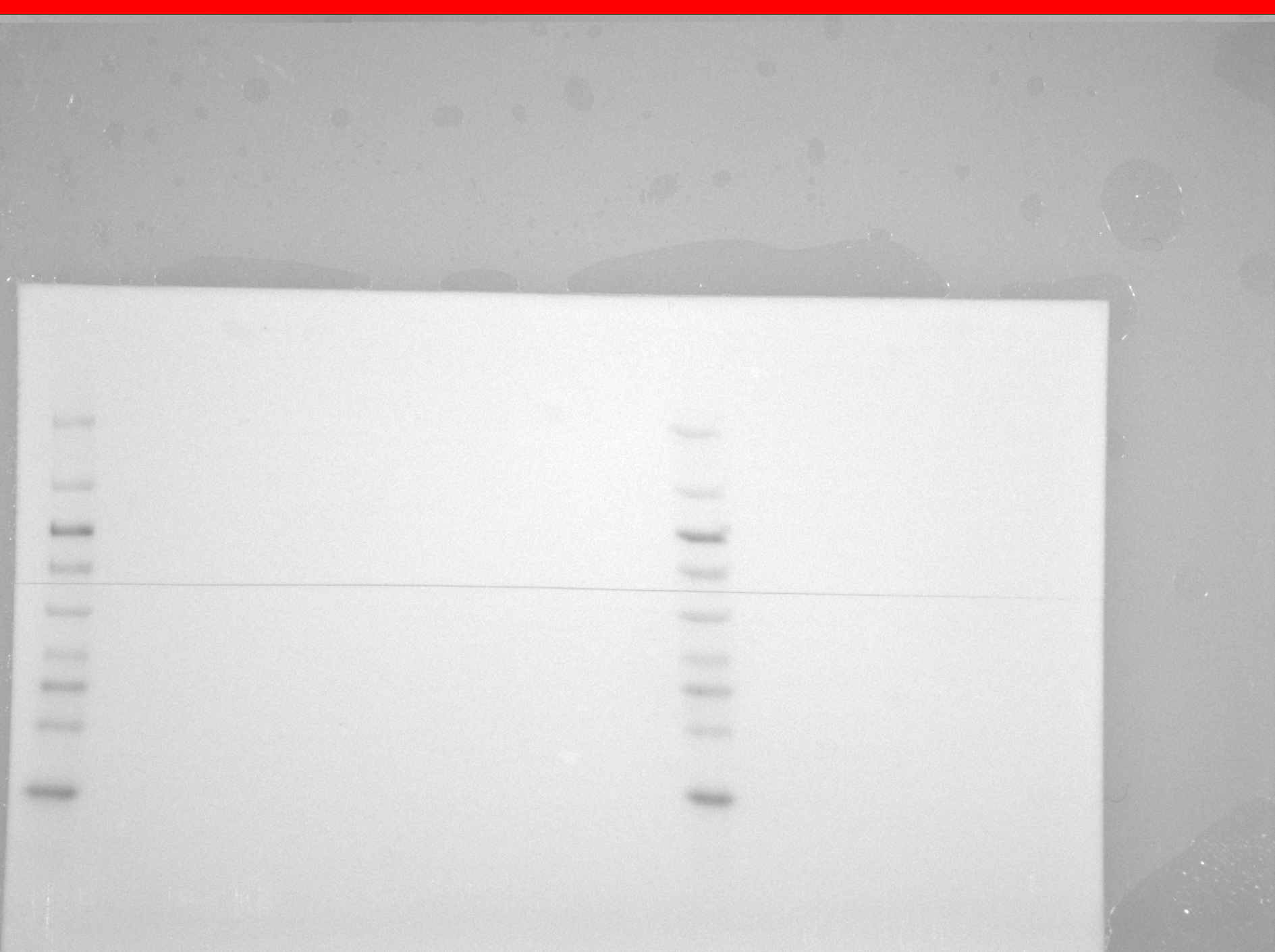

Supplement: Supplementary file 6 — Unedited western blot images. [file 41586_2024_8455_MOESM6_ESM.zip › Raw western images/2024-01-18-8.tif]
